# G-Protein Coupled Receptor Signaling Architecture of Mammalian Immune Cells

**DOI:** 10.1371/journal.pone.0004189

**Published:** 2009-01-14

**Authors:** Natalia Polouliakh, Richard Nock, Frank Nielsen, Hiroaki Kitano

**Affiliations:** 1 Sony Computer Science Laboratories Inc., Tokyo, Japan; 2 Systems Biology Institute, Tokyo, Japan; 3 CEREGMIA- Univ. Antilles-Guyane, Schoelcher, France; Dresden University of Technology, Germany

## Abstract

A series of recent studies on large-scale networks of signaling and metabolic systems revealed that a certain network structure often called “bow-tie network” are observed. In signaling systems, bow-tie network takes a form with diverse and redundant inputs and outputs connected via a small numbers of core molecules. While arguments have been made that such network architecture enhances robustness and evolvability of biological systems, its functional role at a cellular level remains obscure. A hypothesis was proposed that such a network function as a stimuli-reaction classifier where dynamics of core molecules dictate downstream transcriptional activities, hence physiological responses against stimuli. In this study, we examined whether such hypothesis can be verified using experimental data from Alliance for Cellular Signaling (AfCS) that comprehensively measured GPCR related ligands response for B-cell and macrophage. In a GPCR signaling system, cAMP and Ca^2+^ act as core molecules. Stimuli-response for 32 ligands to B-Cells and 23 ligands to macrophages has been measured. We found that ligands with correlated changes of cAMP and Ca^2+^ tend to cluster closely together within the hyperspaces of both cell types and they induced genes involved in the same cellular processes. It was found that ligands inducing cAMP synthesis activate genes involved in cell growth and proliferation; cAMP and Ca^2+^ molecules that increased together form a feedback loop and induce immune cells to migrate and adhere together. In contrast, ligands without a core molecules response are scattered throughout the hyperspace and do not share clusters. G-protein coupling receptors together with immune response specific receptors were found in cAMP and Ca^2+^ activated clusters. Analyses have been done on the original software applicable for discovering ‘bow-tie’ network architectures within the complex network of intracellular signaling where *ab initio* clustering has been implemented as well. Groups of potential transcription factors for each specific group of genes were found to be partly conserved across B-Cell and macrophage. A series of findings support the hypothesis.

## Introduction

Understanding the logic behind complex mammalian signaling networks is both a scientifically and medically significant issue. Recent efforts to depict signaling networks using extensive experimental techniques have begun to reveal the nature of some of these signaling networks [1]–[5]. These studies typically use a variety of ligand stimuili and measure cellular responses, such as changes in gene expressions, the phosphorylation of proteins in the signaling networks, changes in second messengers, and cellular physiology, such as secretions of cytokines and apoptosis. Statistical methods like principal component analysis are often used to identify the principal contributing features and possible novel interactions. This is a data-driven approach and has proven to be effective. A rich data set from Alliance for Cellular Signaling (AfCS) provides furtile ground for extensive analysis to depict logics behind cellular signaling. Studies published to date focused on a clustering-based approach to identify salient correlation between stimuli and gene expressions, discovery of possible unknown interactions, and identifications of key molecules for signaling processes [1], [5], [6].

**Figure 1:**
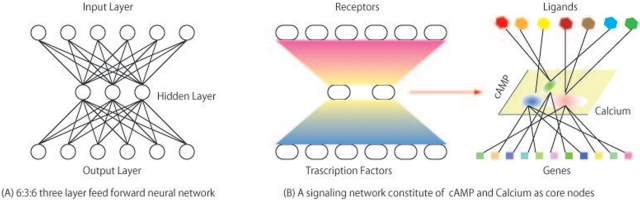
Bow-tie network for signaling. Bow-tie networks generally have diverse inputs and outputs with conserved core nodes. It is called “bow-tie” as its pictorial representation resembles bowtie. Fig 1(a): In a three layer feed forward neural networks, a hidden layer (a middle layer) provides generalization capability and the neural network can function as classifier of diverse inputs mapping into diverse output patterns. It is essential that numbers of nodes in the hidden layer is smaller than numbers of nodes in inputs and output layers in order to achieve high level of generalization. This is an example of 6∶3∶6 feed forward network with 6 input nodes, 3 hidden nodes, and 6 output layer nodes. Fig 1(b): In signaling networks, receptors corresponds nodes in an input layer, molecules such as cAMP and calcium corresponds nodes in a hidden layer, and transcription factors corresponds to nodes in an output layer (Left). Looking at this from a stimuli-response viewpoint, it shall be viewed as a process that diverse ligands activate variety of receptors forming distinct activation patterns and results in patterns of activations at transcriptional level (Right). An intermediate layer (core nodes) that corresponds to the hidden layer in the feed forward neural network shall provide generalization capability to the signaling network. In GPCR pathway, cAMP and calcium are key molecules constituting this layer. Thus, diverse stimuli are classified into several groups that have similar calcium and cAMP elevation. Ligands that are classified into the same group shall activate similar subset of genes, hence invoking similar physiological responses, if generalization is actually taking place.

In this work, we took a different approach. We used clustering analysis to examine whether “bow-tie” architecture of signaling network plays any functional role in cellular signaling and, if so, what role does it play. “Bow-tie” network is a kind of networks that its pictorial representation often resembles a bow tie and its concept is shown in Figure 1. It consists of sub-networks with diverse inputs converting into a conserved core sub-network (an input wing), another conserved core sub-network (a bow-tie core), and an output sub-network that enables diverse responses to the input stimuli (an output wing). There have been an increasing number of reports on a “bow-tie” network architecture in metabolic and signaling networks [7]–[9], and arguments have been made that this is a critical feature of robust yet evolvable systems [11], [12] that can be also applied for network structure of the Worldwide Web[13]. Studies of metabolic network structures in bacteria [8] and in human [7], inter-cellular communications in immune system [1], epidermal receptor signaling networks [10], and Toll-like receptor signaling networks [9] demonstrates such networks seems to exists in various aspects of biological systems.

It was noticed that the structure of the bow tie networks found in metabolic and signaling networks are very different. In a metabolic network, the core forms a giant cluster, where the nodes are densely interconnected. On the other hand, the bow tie network found in signaling networks has fewer nodes with sparse interconnections even if such connections exist. Naturally, the roles of the cores in metabolic and signaling networks differ. In metabolic networks, the core provides a robust central processing factory where various nutrients flow in and produce ATP, amino-acids, and other essential metabolites [8], [11]. The question is: what is the functional role of the core in signaling networks?

A hypothesis has been proposed that claims that small numbers of molecules in the core of bow tie signaling networks may constitute an evolutionary acquired learning layer that takes on various stimuli, generalizes the stimuli into a few separate classes, and relays them to transcription factors [2]. This hypothesis was inspired by neural network research that indicates that the generalization and learning of various stimuli-reaction is best accomplished when there are fewer middle layer nodes than input and output layers in three-layer feed-forward networks, because middle layers with limited nodes are forced to generalize the information to accomplish accurate reactions for a broad range of stimuli [14]. Given the similarity in network structures, although signaling networks are more complicated and less organized, it is reasonable to ask the question of whether similar phenomena in the generalization capacity can be observed in the core of signaling networks. In other words, we can test the hypothesis that nodes in the core of a bow tie network form a classifier of reactions against stimuli are predictable if the dynamics of such molecules are observed. This question is both scientifically and practically significant because it not only depicts the logic behind the network architecture, but also helps us uncover the potential control points of signaling networks for drug design.

In a GTP-coupled protein receptor (GPCR) signaling network, calcium and cAMP are considered to be the nodes in the core of a bow tie network in which a variety of signals from the GPCR converge and are relayed downstream of the network. Previous works using clustering approach on AfCS data also argue critical role of calcium and cAMP [5], [6]. Therefore, the hypothesis can be tested by investigating following two points. First, whether ligand induced dynamics Ca^2+^ and cAMP can form distinct clusters that categorize the ligands into corresponding clusters. Second, can behaviors of ligand induced dynamics of calcium and cAMP predict which groups of genes may be up-regulated by the stimuli.

In order to test this hypothesis, a publicly accessible open dataset from the Alliance for Cellular Signaling (AfCS) [3] has been used for this study. Both B-cell and macrophage datasets were used in the analysis of this paper. The AfCS dataset enabled us to cluster the calcium elevation and cAMP production into four clusters, and each cluster corresponds to a group of genes involved in distinct cellular processes. A transcription analysis revealed that the transcription factors activated for each Calcium-cAMP cluster are partly conserved between B-cells and macrophages.

## Results and Discussion

### Data

A publicly available data set from the Alliance for Cellular Signaling (AfCS) was used for analysis in this paper. In particular, a set of expression profile data, calcium level data, and cAMP level data for single ligand assay in both B-cells and macrophages was used. Expression profiles data for 32 B-Cell ligands (0.5, 1, 2, and 4 h) and 5 macrophage ligands (1, 2, and 4 h) was available. In the case of B-Cells, 2937 differentially expressed features (feature were cDNA) [5] were used. We then selected features using log(treated/control) ≥0.2 and ≤−0.2 for the macrophage, and 778 features were obtained for clustering.

### Ligands grouping by cAMP and Ca^2+^ response to stimuli

Ca^2+^ and cAMP are elevated within a cell upon stimuli, and the level of elevation can be classified into several clusters depending upon the combinations of elevation levels. The degree of elevation of Ca^2+^ and cAMP upon ligand stimuli has been mapped onto a two-dimensional Ca^2+^-cAMP space. When a group of ligands stimuli elevate Ca^2+^ and cAMP to a similar level, these ligands are mapped onto closer within the hyperspace and may be categorized as a *sub*-region. Thus, Ca^2+^ and cAMP elevations by different groups of ligands are mapped into different *sub*-regions within the network space and a conceptual view of this is represented in Figure 1.

**Figure 2:**
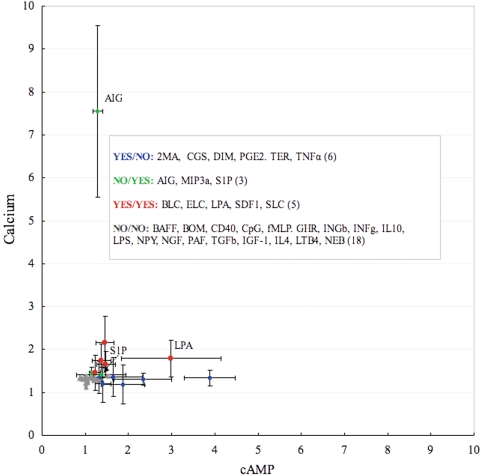
Peaks of fold changes for 32 B-Cell ligands classified into four groups with observed ‘YES’ and non-observed ‘NO’ elevation of cAMP and Ca^2+^. Standard deviations (SD) are shown by the vertical bars. Full names for the ligands are listed in Table S1. S1P and LPA ligands are marked to be visible as they are given more detailed discussion in the manuscript.

**Figure 3:**
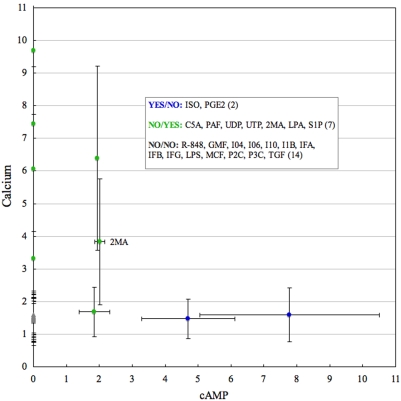
Peaks of fold changes for 23 macrophage ligands classified into three groups with observed ‘YES’ and non-observed ‘NO’ elevation of Ca^2+^ and cAMP. Standard deviations (SD) are shown by the vertical bars. Full names for the ligands are listed in Figure S2. Values equal to zero for cAMP correspond to the experimentally uncharacterized cAMP. 2MA ligand is marked to be visible as it is given more detailed discussion in the manuscript.

To attribute these differences to the levels of elevation, we used ‘YES’ nomenclature to indicate elevated states and ‘NO’ to indicate those that were non-elevated. An increase in cAMP synthesis or Ca^2+^ mobilization when ligands were added was assigned ‘YES’, and an unchangeable state was assigned ‘NO’. Thus, 32 ligands for B-Cell ligands and 23 macrophage ligands were simply classified into four groups: ‘YES/NO’, ‘NO/YES’, ‘YES/YES’, and ‘NO/NO’, where the former refers to the cAMP state and the latter specifies the Ca^2+^ status. The ‘YES’ and ‘NO’ assessments that were conducted based on the experimental annotations provided with the data and the ligands mapped on the different *sub*-regions on two cells are shown in Figures 2 and 3. Ligands names are listed in Supplementary Tables S1 and S2. From Figure 2 we can notice that although S1P was annotated as a Ca^2+^ - inducing ligand it has cAMP level similar to Dimaprit, and according to the clustering results was further considered a candidate for the ‘YES/YES’ group. Lysophosphatidic acid (LPA) induced cAMP stronger than Ca^2+^ and Anti-Ig (AIG) was the top Ca ^2+^-inducing ligand.

### cAMP and Calcium-induced *sub*-regions control proliferation and chemotaxis of B-Cell

We avoided clustering together NO/NO, YES/NO, YES/YES and NO/YES groups, and clustered NO/NO group separately. The reason was that NO/NO group included 4 ligands (CD40, LPS, CpG, IL4) with the strongest effect on gene expression that are unrelated to cAMP and calcium, and their simultaneous clustering with YES/NO, YES/YES and NO/YES groups could shade meaningful changes in the expression induced by other ligands. By the same reason the Anti-Ig (AIG) strongest NO/YES ligand was analyzed in correlation with NO/NO ligands, with which it had the most similarity shown by the previous study [5].

**Figure 4:**
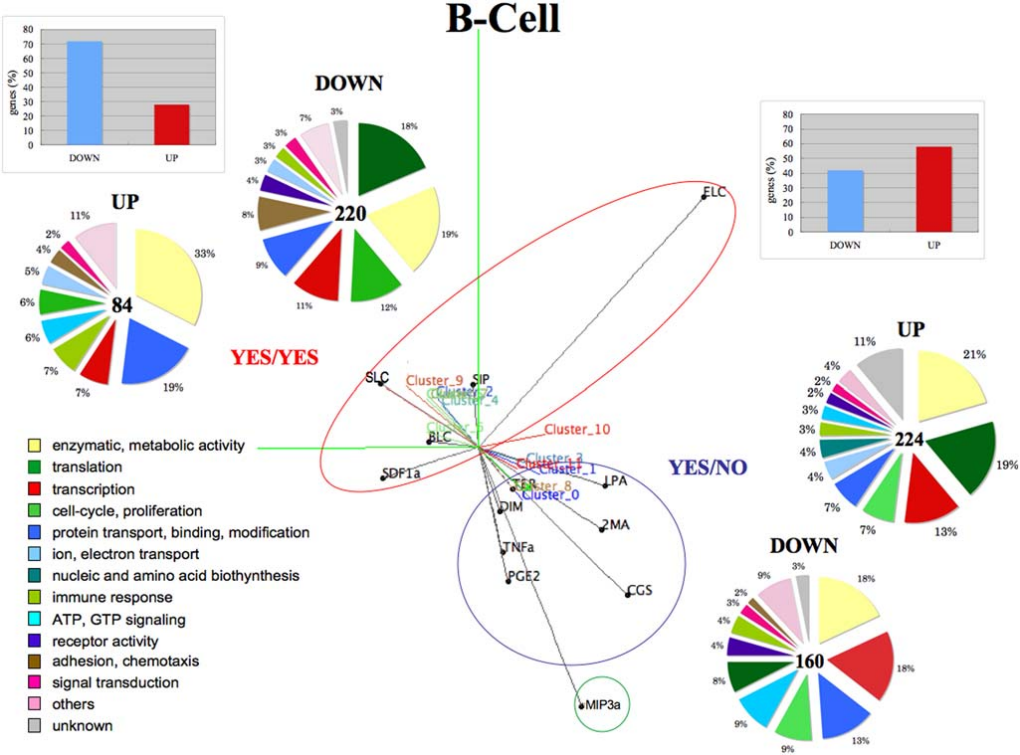
Clustering results for YES/NO (cAMP), YES/YES (cAMP-Ca^2+^) and NO/YES (Ca^2+^) groups. Ligands group into two big *sub*-regions: YES/NO and YES/YES and one small *sub*-region represented by MI3a (NO/YES) ligand. The M3A ligand mainly induced by Ca^2+^ is separated and does not form a cluster downstream. This representation, which we call ‘PCA correlation ball’, shows both the groups of ligands and their correlations, and also the regulations of gene clusters from the projections of nearby ligands. Bow-graphs in the upper corners show the percent of genes being *up-* or *down-* regulated after the expiration of one/both molecules activation limited to first time marks (30 min in B-Cell), and the differences are obvious. Genes functions (‘Function’ or ‘Cellular process’ (GO terms), if ‘Function’ is absent) calculated basing on the enrichment in clusters with p-value<0.05 are shown in the pie-graphs.

The clustering results for the YES/NO, YES/YES and NO/YES groups of the B-Cell ligands are shown in Figure 4. PCA correlation balls are used to plot in three dimensions both ligands, like in conventional correlation circles for PCA, but also clusters' principal components (see Method). This shows both the groups of ligands and their correlations, and also the regulations of gene clusters from the projections of nearby ligands.

We observe ligands that separate into two big *sub*-regions facing the opposite directions, which included YES/NO (cAMP) and YES/YES (cAMP-Calcium) ligands, respectively. MIP3a and S1P, both categlized into NO/YES ligands group, did not form a distinct *sub*-region. This might be explained that S1P has cAMP level similar to YES/NO ligands group, although it was annotated as Ca^2+^ -activating. MIP3a did not form an independent downstream cluster.

All the ligands were quite closely located in the YES/YES *sub*-region except for ELC. It is interesting to find LPA in the YES/NO *sub*-region rather than in the YES/YES *sub*-region, which it was classified in, but by looking at Figure 2, we can see it having the strongest peak for cAMP compared to the other ligands in YES/YES group. These two big groups of genes included 675 genes for the YES/NO *sub*-region and 680 for the YES/YES *sub*-region. It was interesting to find that 70% of the genes in the YES/NO clusters were *up*-regulated and 80% of the genes in the YES/YES clusters are suppressed. This means that the termination of activation of cAMP or/and Ca^2+^ promoted the deactivation of 30% of the genes in the YES/NO *sub*-region and 80% of the genes in the YES/YES *sub*-region. Based on this, we can consider the combination of cAMP- Ca^2+^ to be two times more suppressive, than cAMP alone.

We calculated the functional enrichment of genes in the *up*-regulated and *down*-regulated clusters using the chi-square test basing on gene ontology (GO) annotations and depicted the genes with a p-value<0.05 in the pie-graphs functional categories of Figure 4. The biological meaning of the YES/YES *sub*-region can be related to the fact that 3 of the 6 ligands, such as BLC, SLC, and ELC, induce the localization and migration of B-Cells to the secondary lymphoid tissue and SDF1 retains the B-Cell in the bone marrow during B-Cell development [3].

We have found more genes involved in chemotaxis, cell-adhesion, and cytoskeleton reorganization, for example, Appbp2, Col14a1, Cdh11, Daam1, Lamc1, Tmsb10, and Tnfrsf12a (12% of genes categorized to be involved in chemotaxis, cell-adhesion, and cytoskeleton using Gene Ontology), comparing to YES/NO sub-region (2% of genes). This supports our conjecture that YES/YES *sub*-region is related to cell-trafficking and adhesive function. Although 8% of genes out of 12% are *down*-regulated to 4 hours, activation of them at the early stage contributes their functional role. It has been reported [15] that intracellular calcium levels can depolarize the plasma membrane and regulate B-Cell adhesion and trafficking independent of the expressed B cell receptor (BCR).

There are also more genes involved in translation (24%) than in transcription (18%) and in larger groups of immune response genes (10%).

The YES/NO *sub*-region is notable for containing genes involved in cell-proliferation (16%), transcription (31%), translation (27%) and also genes involved in GTP-ase induced signaling and cAMP biosynthesis (12%), and Atp6v1f, Adcy5, Gnai2, Rgs14, Gnb2-rs1, Grap, Prkcn, Rangap, Rsu1, 2810441C07Rik are examples that do this. This region also has 13% higher inclusion of enzymes (52%) compared to the YES/NO *sub*-region (39%).

Receptors of YES/NO and YES/YES *sub*-regions of B-Cell are listed in Table 1. Although there were a few, we have 3 G-protein coupled receptors (GTP-bindng) and several immune response receptors.

**Table 1:** Receptor genes expressed in YES/NO and YES/YES *sub*-regions of B-Cell.

Receptor ID	Full name	Group
**Fzd4**	Frizzled homolog 4, GPCR	YES/NO
**Ssr1**	Signal sequence receptor, alpha, GTP-binding	YES/NO
**Igl-V1**	Immunoglobulin lambda chain, variable 1	YES/NO
**Gabbr1**	Gamma-aminobutyric acid (GABA) B receptor 1	YES/NO
**Olfr701**	Olfactory receptor, GPCR	YES/YES
**Ahr**	Aryl-hydrocarbon receptor (cell-cycle, apoptosis)	YES/YES
**Tnfrsf12a,13c**	Tumor necrosis factor receptor superfamily (adhesion, apoptosis)	YES/YES

### NON-responding ligands and Anti-Ig

We checked how tight the correlations are among the NO/NO ligands when they are clustered together and the results are shown in Figure S1. Although we found a couple of pairs where ligands map quite closely (fMLP-NGFb, LPS-CpG), the ligands in this group looked much more disconnected than in the other two groups.

**Figure 5:**
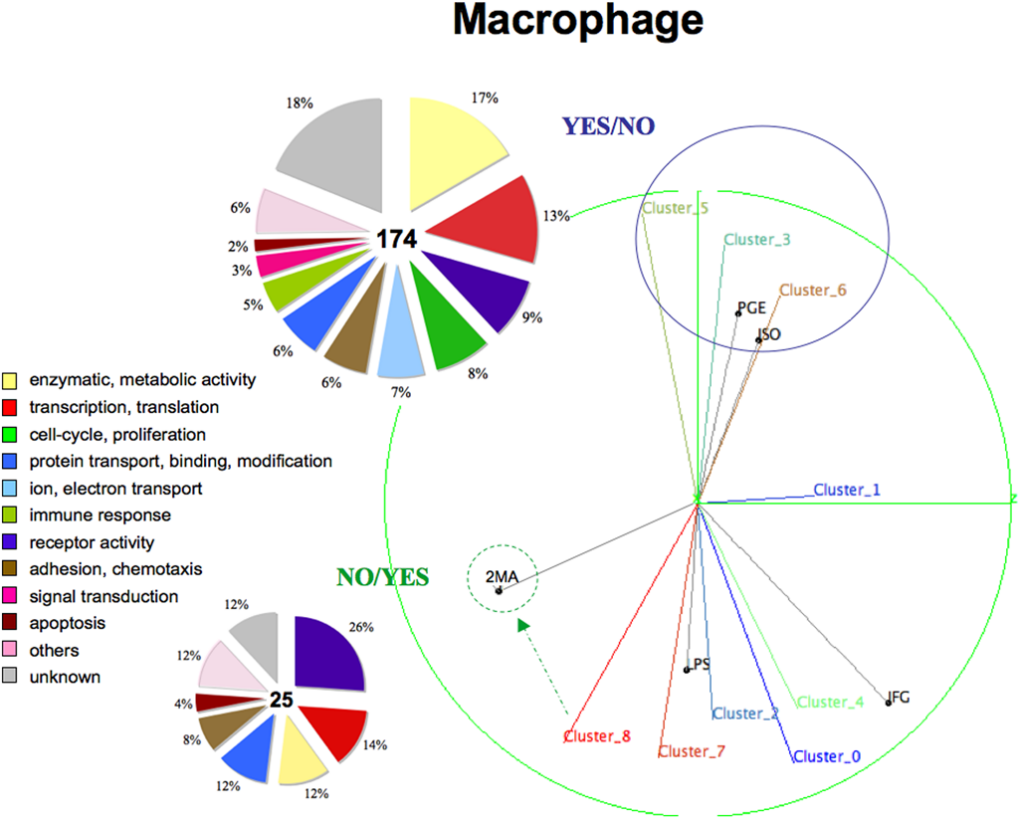
Clustering results on 5 macrophage ligands with available microarray expression data: PGE, ISO (YES/NO), 2MA (YES/YES) and LPS, IFG (NO/NO). Two cAMP-activated ligands (PGE and ISO) form a distinct *sub*-region within the hyperspace. NO/NO ligands (LPS and IFG) do not form a single *sub*-region. 2MA is an only YES/YES ligand remoted from YES/NO *sub*-region as well as from NO/NO ligands. Possibly Cluster_8 is the influenced by 2MA. This representation, which we call ‘PCA correlation ball’, shows both the groups of ligands and their correlations, and also the regulations of gene clusters from the projections of nearby ligands. Genes functions (‘Function’ or ‘Cellular process’ (GO terms), if ‘Function’ is absent) calculated basing on the enrichment in clusters with p-value<0.05 are shown in the pie-graphs. By the reason of small number of genes we combined *up-* and *down-* regulated gene together in the pie-graphs.

Next, we added AIG to the NO/NO group and clustered genes. AIG is the strongest Ca^2+^ ligand and we observed its strong correlation with CD40, LPS, CpG and IL4 (Figure S2). These clustering results on these 5 ligands are identical to the finding uncovered in paper published on original data [5]. It is interesting to find AIG (Anti-Ig) NO/YES ligand close to LPS in B-Cell (Fig. S1) and 2MA NO/YES ligand sharing Cluster_8 with LPS in macrophage (Figure 5).

### YES/NO *sub*-region in macrophage clusters separately

All the macrophage ligands were clustered together as there were only a few (5 ligands). Isoprenol (ISO) and Prostaglandin 2 (PGE2) after cAMP induction can be seen clustered closely together in Fig. 5. 2MA, which is only a NO/YES (Ca^2+^) ligand, is located away from the YES/NO and NO/NO ligands (LPS, IFG). We have considered possibly inducing Cluster_8 together with LPS. The YES/NO *sub*-region was found to be interesting and had similarities with B-Cell, such as possessing many transcriptions (13%), GTP-ase (9%), proliferation (8%), and ion transport (7%) genes. It also had a group of enzymes (17%), but they were still smaller than that in the YES/NO group of B-Cell. What was most remarkable was the set of receptors involved in the YES/NO and NO/YES groups (Table 2). Half of them were G-protein coupled receptors (Ptger2, Avpr1b, Ccr1, Ccrl2, F2rl2, etc.) and the other half were involved in the immune response (Igk-V1, Il7r, Cd16 Cr2, Tnfrsf1b). Although we do not separately show the *up*- and *down*-regulated clusters because we had only comparatively small data, the expression profiles of GPCRs were similar and the genes lost their expression activities after termination of cAMP. We can assume that the GPCRs activation is as important for the proliferation program of cells [16], [17], [18], [19] as cAMP itself. We also have checked the receptors scattered around the NO/NO ligands and found two distinct groups: GPCRs (F2rl3, Htr2b, Lgr5, & Ltb4r1) suppressed more than two folds at the first time mark and immune response receptors (Toll-like receptor, Natural killer cell receptor, and Xenotropic retrovirus receptor) gradually activated.

**Table 2:** Receptor genes expressed in YES/NO and NO/YES *sub*-regions of macrophage.

Receptor ID	Full name	Group
**Ptger2**	Prostaglandin E receptor (subtype EP2), GPCR	Y/N
**Avpr1b**	Arginine vasopressin receptor 1B, GPCR	Y/N
**Ccr1**	Chemokine (C-C motif) receptor 1, GPCR	Y/N
**Ccrl2**	Chemokine (C-C motif) receptor-like 2, GPCR	Y/N
**F2rl2**	Coagulation factor II (thrombin) receptor-like 2, GPCR	Y/N
**Gpr146**	G-protein coupled receptor, GPCR	Y/N
**Olfr948**	Olfactory receptor, GPCR	Y/N
**Vmn2r89**	Vomeronasal 2, receptor 89, GPCR	Y/N
**Igk-V1**	Immunoglobulin kappa chain variable 1 (V1)	Y/N
**Il7r**	Interleukin 7 receptor	Y/N
**Cd160**	CD160 antigen, MHC class I	Y/N
**Cr2**	Complement receptor 2	Y/N
**Tnfrsf1b**	Tumor necrosis factor receptor	Y/N
**Nr4a2**	Nuclear receptor subfamily 4, group A, member 2	Y/N
**Gabra1**	Gamma-aminobutyric acid A receptor, alpha 1	Y/N
**Brs3**	Bombesin-like receptor 3, GPCR	N/Y
**Gpr84**	G-protein coupled receptor, GPCR	N/Y
**Il5ra**	Interleukin 5 receptor, alpha	N/Y
**Cd22**	CD22 antigen (adhesion)	N/Y
**Cd244**	Natural killer cell receptor 2B4	N/Y
**Gosr1**	Golgi SNAP receptor complex member 1	N/Y

### Transcription regulation analysis in B-cell and macrophage

Given these differences in genes that are activated in each ligands group that are categorized based on the sub-regions on the cAMP-Calcium space, a next natural question to ask is what transcriptional factors are involved in the expression of genes in different sub-regions and how specific are they? We do not use phylogenetic footprinting because the downstream signaling in immune cells can be weakly conserved in higher eukaryotes, and therefore only mouse data has been used for analysis.

### Finding *cis*-elements

For the analysis we used the F-match software within ExPlain2.4 package [20], [21]. F-match searches against the largest library (>500) of position specific scoring matrices (PSSM) compiled on experimentally verified transcription factors binding sites of the higher eukaryotes [20], [21] from the TRANSFAC database [20].

F-Match evaluates the set of promoters and for each matrix tries to find two thresholds: one, *th-max*, which provides maximum ratio between the frequency of matches in the promoters in focus (control set ‘C’) and background promoters (background set ‘B’) (over-represented sites); and the second threshold, *th-min*, that minimizes the same ratio (underrepresented sites). A binomial distribution of the sites between two sets is calculated and the p-value is assigned to the probability that the observed number of sites and higher, for over-represented matches, or lower, in the case of under-represented matches.

Sequences to 2000 bp upstream and 500 bp downstream from the transcriptional start sites (TSS) of the integrated TRANSPro database (ExPlain2.4) accumulating TSS evidences from EPD, DBTSS, and Ensembl databases were used for the analysis.

### Comparison between YES/NO (cAMP) and YES/YES (cAMP-Calcium) groups in B-Cell

As we had enough data to separate genes in YES/NO and YES/YES groups into Up-regulated (UP) and Down-regulated (DOWN) *sub*-groups (Fig. 4) the biggest interest for us was to find differences in the transcription regulators of those *sub*-groups. Thus we did search having following pairs for the control[C] and background [B] sets: YES/NO(UP)[C] to YES/YES(UP)[B] and vice-versa, and YES/NO(DOWN)[C] to YES/YES(DOWN)[B] and vice-versa. Finally we had 4 resulted sets of possible candidates for transcription factor binding sites (TFBS) represented by the TRANSFAC matrices names. We picked up top 30 TFBS for each group, excluded matrices of qualities 5 and 6 (‘_Q5’, ‘_Q6’) and plotted the rest of them as possible candidates for the transcription factors binding (Figure S3).

We identified 18 TFBS being shared in different combinations by the promoters of genes in 4 groups of B-Cell (YES/NO‘UP’, YES/NO‘DOWN’, YES/YES‘UP, YES/YES‘DOWN’ and group-specific TFBS.

23% (25/78) of TBFS are known to be Immune System regulators, such as, AP1, GATAs, PPARA, MZF1, IK1, TAL1ALPHA(BETA)E47, CEBP, NFKAPPAB50, EGRs, etc [22], [23].

### Comparison between YES/NO (cAMP) and NO/YES (Calcium) groups in Macrophage

In the case of macrophage we did not separate the genes into Up- and Down-regulated sub-groups in macrophage, as we had a very small and putative dataset (Cluster_8) for the NO/YES *sub*-region of the macrophage to fairly compare them. We compared YES/NO[C] group against NO/YES[B] as a background and vice-versa, and the results are shown in Figure S4. We found the same percentage 23% (9/39) of known Immune System regulators and only 4 TFBS were shared by YES/NO and NO/YES groups. It would be interesting if this could explain the stronger differences between the YES/NO and NO/YES datasets, which do not share a common molecule, than in the YES/NO and YES/YES datasets, which share cAMP. *Sub*-region specific TBFS candidates were observed with p-values slightly lower than in B-Cell. Macrophage and B-Cell (Up- and Down- together) YES/NO groups have common TFBS, which are MZF1, CEBP, AP2ALPHA, GR1, AR, SRY, etc.

In case we compare the sets of TFBS found in B-Cell and Macrophage 21% (24 out of 113 unique names) are common, although some bias from the dataset sizes could be expected.

By taking into account the similarities and differences described above, we concluded that some combinatorial effect of the transcription factors together with specific groups of the transcription factors are utilized in regulating *sub*-regions formed by the different statuses of activation of cAMP and calcium. Although some bias is expected from the different background dataset sizes, we hope that our findings will help in the further investigations on the transcription regulation network in immune cells. In this work our purpose was limited by the discovery of the possible transcription factor candidates with combinatorial and group-specific destinations within the bow-tie signaling network architecture.

As a result of our analysis, the correlation between the ligand groups and the groups of genes that are activated is obvious, and the *sub*-regions within the cAMP-Calcium space bridges the ligand groups and genes that are to be activated in each stimuli. Ligands that are categorized in the YES/NO group promote the general proliferation of cellular activities and the YES/YES group tends to activate the adhesion and migration of related genes. These two groups include many GPCRs as well as nuclear and immune system-specific receptors. The NO/NO ligands do not produce meaningful downstream clusters and few GPCRs found in macrophage are strongly *down*-regulated by these ligands, whereas the immune response receptors were found continuously *up*-regulated. Thus, we believe the hypothesis is likely correct, and should be considered to be one of the logics behind signaling systems. The investigation into and further elaboration of such a study is warranted to determine if there are additional molecules that can further sub-categorize cellular responses so that the detailed differences in cellular responses can be explained at a network architecture level.

## Methods

### Clustering ligands and genes

The clustering analysis was carried out on ‘in house’ software especially developed for the task. This task consists in grouping genes in clusters, according to the similarity of their measurements; we performed a hard membership clustering, in which each gene belongs exactly to one cluster. However, rather than directly performing clustering, we chose to first preprocess data: it is indeed widely accepted that clustering results are often improved when one plunges data in a low-dimensional space which captures the intrinsic manifold on which they lie [24], [25], [26]. Overall, we get five steps for preprocessing and clustering. First, a time series for gene i and ligand j was mapped to a slope s_ij_ using a conventional linear regression fit:
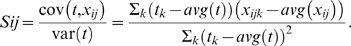
Here, “avg” denotes the average, “var” is the variance and “cov” is the covariance; furthermore, *t* denotes the set of time stamps and *x_ij_* denotes the set of measurements for gene *i* and ligand *j*. *k* spans {0, 1, 2, 3} (B-Cell) and {0, 1, 2} (Macrophage). Since only two measurements are necessary for one slope, we do not need to discard genes with incomplete sets of measurements. Second, a similarity matrix M is computed, whose entry in row *i*, column *l* (*m_il_*) is the similarity between gene *i* and *l*, chosen to be proportional to the heat kernel [24]:
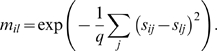
Here, q>0 is a real parameter to be fixed by the user (q = 2 in our experiments). Matrix M is then post-processed following a procedure close to Local Linear Embedding [25]. The user chooses an integer r>0 (r = 5 in our experiments); for each row i of M, we search for the r largest entries whose columns define the r nearest neighbors of gene i. For each row i of M, we then compute the symmetric nearest neighbors of each gene i, by aggregating both the nearest neighbors of the gene, and the genes for which gene i is a nearest neighbor [27]. We finally replace M by using the Boolean indicator matrix for symmetric nearest neighbors (m_il_ = 1 if genes i and l are symmetric nearest neighbors, or 0 otherwise). Matrix M remains symmetric, and turns out to be a very convenient input for the third step, which seeks the closest doubly stochastic approximation of M [26]. This doubly stochastic approximation finds the solution to the following problem:
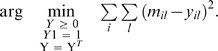
The first condition on Y is non-negativity; with the second condition, Y is a Markov chain's transition matrix. With the last condition, Y becomes doubly stochastic. In the fourth step, the spectral decomposition of Y is computed, and we seek the leading d non-trivial eigenvectors of this decomposition, which yield the new genes coordinates in 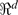 [26]. There is no universally efficient rule of thumb to choose d; we decided to pick d = 3, as this makes clustering fit to the representation of genes in three dimensions. We also noted that this choice was accurate from the standpoint of the eigenvalues, as the first three stood in general significantly above the others.

The fifth and last step is a conventional k-means algorithm on these new manifold coordinates. We have run numerous experiments for different values of k, and kept the value of k that visually yields the strongest bend in k-means potential and k-means intracluster kernel similarities. The clusters are visualized in this paper on three-dimensional PCA correlation balls, instead of two-dimensional correlation circles. Each cluster u is represented by ***c*** *_u_*, the expectation of its members manifold coordinates, i.e.:
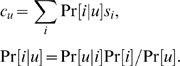
Here, s_i_ is the vector description of gene i (each coordinate is a slope); Pr[u|i] is the membership probability for gene i in cluster u, i.e. the indicator variable for the membership in cluster u, known from the clustering results; Pr[i] is the probability of gene i, known since it is the number of replica measurements for the gene over the total number of replica measurements for all genes; finally, Pr[u] is the probability of cluster u. This last probability is unknown, so we end up with an approximation of c_u_ of the form:
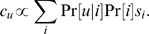
Rather than plotting a point, we plot a line segment whose direction is given by the right-hand side of the preceding equation.

## Supporting Information

Table S1 Classification of 32 B-cell ligands into 4 groups according to their cAMP and Ca2+ fold increase by the expertise provided with the data. ‘YES’ was assigned to the induced state and ‘NO’, otherwise. The former annotation refers cAMP and the latter Ca2+ molecules. The respective numbers of the ligands are: ‘YES/NO’ - 6, ‘NO/YES’ - 3, ‘YES/YES’ - 5 and ‘NO/NO’ - 18.
(0.07 MB DOC)

Table S2 Classification of 23 macrophage ligands into 3 groups according to their cAMP and Ca2+ fold increase by the expertise provided with the data. ‘YES’ was assigned to the ‘induced’ state and ‘NO’, otherwise. The former annotation refers cAMP and the latter Ca2+ molecules. The respective numbers of the ligands in 4 groups are: ‘YES/NO’ - 2, ‘NO/YES’ - 7 and ‘NO/NO’ - 14.
(0.05 MB DOC)

Figure S1 NO/NO (without cAMP or Ca2+ response) ligands clustered. Except for fMLP-NGF and PAF-IFNb pairs of ligands all ligands this group look uncorrelated, without the explicit projection to the particular downstream clusters.
(5.97 MB TIF)

Figure S2 NO/NO ligands clustered together with AIG NO/YES (strongest Ca2+-inducing ligand). AIG is the closest to CD40, LPS, CpG and IL4 ligands with strong proliferate and differentiation response [5]. It is interesting to notice that adding AIG ligand made the picture of NO/NO ligands completely reorganized, thus brining those 5 ligands closely, while all others moved away.
(5.97 MB TIF)

Figure S3 The common and specific transcription factor binding sites (TFBS) found in 4 sub-groups of YES/NO and YES/YES ligands groups of B-Cell. Horizontal axis lists the matrices names, which former parts (like ‘MZF1’ in MZF1_01) indicate the TFBS name and the latter parts (like ‘_01’ in MZF1_01) point to the experimental-base quality of the matrices in the descending order from ‘1’ to ‘6’. From the top 30 TFBS we selected matrices within the quality index range from 1 to 4. Asterisks* indicate known regulators of the Immune System, which constitutes 23% of the total number of TFBS (25/78) for B-Cell. The respective number of genes in the right-down corner indicates the numbers of genes which promoters were subjected to the transcriptional analysis and depicted in Figure 3 of the manuscript.
(2.53 MB TIF)

Figure S4 The common and specific transcription factor binding sites (TFBS) found in 2 YES/NO and NO/YES sub-regions of macrophage. Horizontal axis lists the matrices names, which former parts (like ‘MZF1’ in MZF1_01) indicate the TFBS name and the latter parts (like ‘_01’ in MZF1_01) point to the experimental-base quality of the matrices in the descending order from ‘1’ to ‘6’. From the top 30 TFBS we selected matrices within the quality index range from 1 to 4. Asterisks* indicate known regulators of the Immune System, which constitutes 23% of the total number of TFBS (9/39) selected for macrophage. The respective numbers of genes in the right-down corner indicate the numbers of genes which promoters were subjected to the transcriptional analysis and depicted in Figure 5 of the manuscript.
(2.37 MB TIF)
