# Supplementary material for: G-Protein Coupled Receptor Signaling Architecture of Mammalian Immune Cells
Source: PLoS One. 2009 Jan 14;4(1):e4189. doi: 10.1371/journal.pone.0004189 (PMC2615211; doi:10.1371/journal.pone.0004189)
Supplement: Table S1 — Classification of 32 B-cell ligands into 4 groups according to their cAMP and Ca2+ fold increase by the expertise provided with the data. ‘YES’ was assigned to the induced state and ‘NO’, otherwise. The former annotation refers cAMP and the latter Ca2+ molecules. The respective numbers of the ligands are: ‘YES/NO’ - 6, ‘NO/YES’ - 3, ‘YES/YES’ - 5 and ‘NO/NO’ - 18. (0.07 MB DOC) [file pone.0004189.s001.doc]

| **Abbrev.** | **Full name** | **Group** |
| --- | --- | --- |
| 2MA | 2-methyl-thio-ATP | YES/NO |
| CGS | CGS-21680 hydrochloride (Adenosine) | YES/NO |
| DIM | Dimaprit | YES/NO |
| PGE2 | Prostaglandin E2 | YES/NO |
| TER | Terbutaline hemisulfate | YES/NO |
| TNFa | Tumor necrosis factor alpha | YES/NO |
| AIG | Anti-immunoglobulin (Ig) | NO/YES |
| MIP3a | Macrophage inflammatory protein-3 | NO/YES |
| SIP | Sphingosine-1-phosphate | NO/YES |
| BLC | B-lymphocyte chemoattractant | YES/YES |
| ELC | MIP-3beta/CCL19 | YES/YES |
| LPA | Lysophosphatidic acid | YES/YES |
| SDF1a | Stromal cell derived factor-1 alpha | YES/YES |
| SLC | Secondary lymphod-tissue chemokine | YES/YES |
| BAFF | B-cell activaing factor | NO/NO |
| BOM | Bombesin | NO/NO |
| CD40L | CD40 monoclonal antibody | NO/NO |
| CpG | CpG-oligodeoxynucleotide | NO/NO |
| fMLP | formyl-Met-Leu-Phe | NO/NO |
| GHRH | Growth hormone releasing hormone | NO/NO |
| IFNβ | Interferon-beta | NO/NO |
| IFNγ | Interferon-gamma | NO/NO |
| IL10 | Interleukin 10 | NO/NO |
| LPS | Lipopolysaccharides | NO/NO |
| NPY | Neuropeptide Y | NO/NO |
| NGFβ | Beta-nerve growth factor | NO/NO |
| PAF | Platelet activating factor | NO/NO |
| TGFβ | Transforming growth factor-beta 1 | NO/NO |
| IGF-1 | Insulin-like growth factor | NO/NO |
| IL4 | Interleukin-4 | NO/NO |
| LTB4 | Leukotriene B4 | NO/NO |
| NEB | Neurokinin beta | NO/NO |
