# Supplementary material for: G-Protein Coupled Receptor Signaling Architecture of Mammalian Immune Cells
Source: PLoS One. 2009 Jan 14;4(1):e4189. doi: 10.1371/journal.pone.0004189 (PMC2615211; doi:10.1371/journal.pone.0004189)
Supplement: Table S2 — Classification of 23 macrophage ligands into 3 groups according to their cAMP and Ca2+ fold increase by the expertise provided with the data. ‘YES’ was assigned to the ‘induced’ state and ‘NO’, otherwise. The former annotation refers cAMP and the latter Ca2+ molecules. The respective numbers of the ligands in 4 groups are: ‘YES/NO’ - 2, ‘NO/YES’ - 7 and ‘NO/NO’ - 14. (0.05 MB DOC) [file pone.0004189.s002.doc]

| **Abbrev.** | **Full name** | **Group** |
| --- | --- | --- |
| ISO* | Isoproterenol hydrochloride | YES/NO |
| PGE2* | Prostaglandin E2 | YES/NO |
| C5A | Complement C5A | NO/YES |
| PAF | Platelet activating factor | NO/YES |
| UDP | Uridine 5’-diphosphate | NO/YES |
| UTP | Uridine 5’-triphosphate | NO/YES |
| 2MA* | 2-methyl-thio-ATP | NO/YES |
| LPA | Lysophosphatidic acid | NO/YES |
| S1P | Sphingosine-1-phosphate | NO/YES |
| R-848 | Resiquimod | NO/NO |
| GMF | Granulocyte macrophage factor | NO/NO |
| IL4 | Interleukin 4 | NO/NO |
| IL6 | Interleukin 6 | NO/NO |
| IL10 | Interleukin 10 | NO/NO |
| IL1b | Interleukin-1b | NO/NO |
| INFα | Interferon-alpha | NO/NO |
| INFβ | Interferon-beta | NO/NO |
| INFγ* | Interferon-gamma | NO/NO |
| LPS* | Lipopolysaccharide | NO/NO |
| MCF | Macrophage colony factor | NO/NO |
| P2C | PAM2CSK4 | NO/NO |
| P3C | PAM3CSK4 | NO/NO |
| TGF | Transforming growth factor | NO/NO |
