# Supplementary figures and images for: G-Protein Coupled Receptor Signaling Architecture of Mammalian Immune Cells
Source: PLoS One. 2009 Jan 14;4(1):e4189. doi: 10.1371/journal.pone.0004189 (PMC2615211; doi:10.1371/journal.pone.0004189)

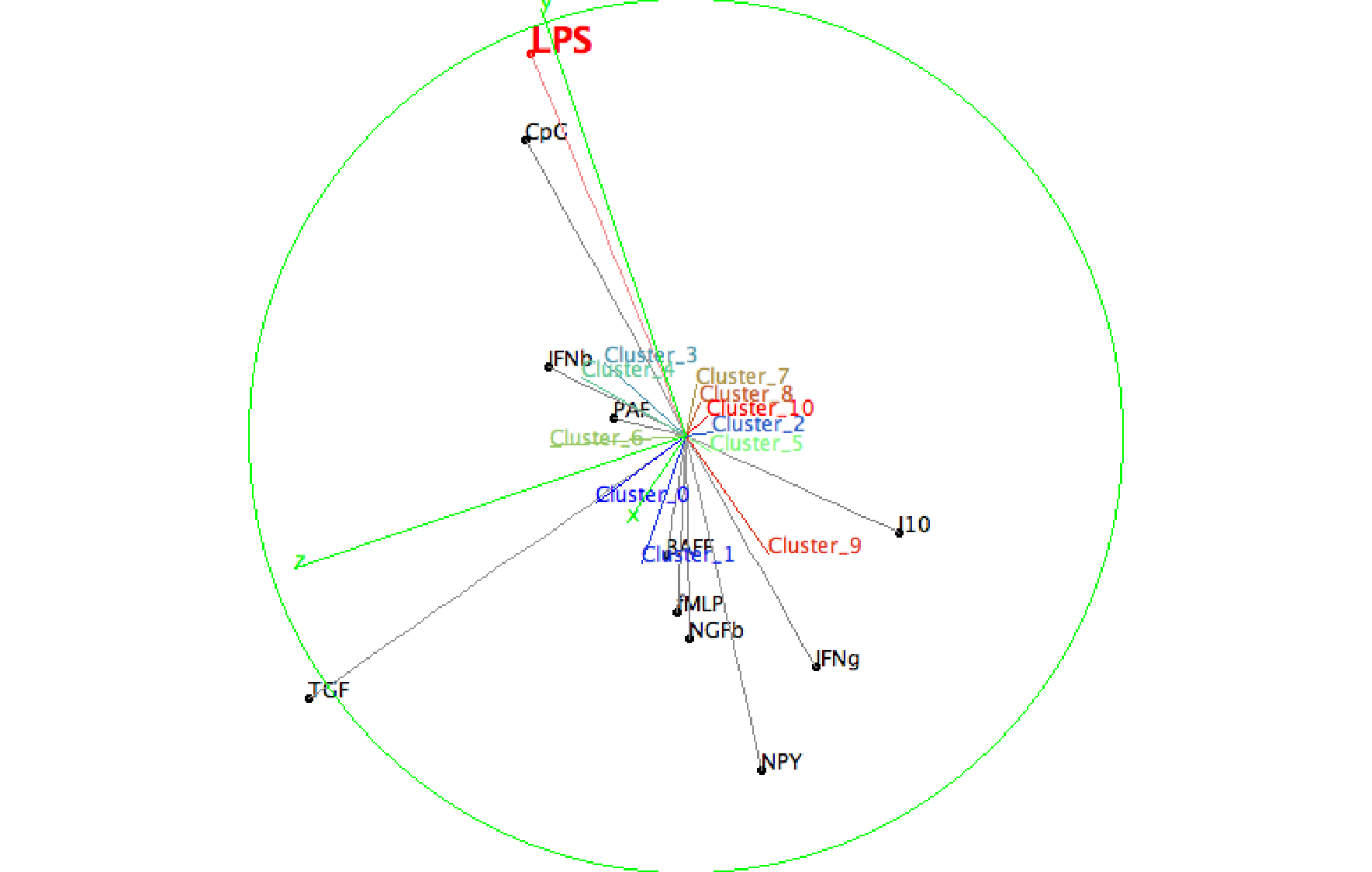

Supplement: Figure S1 — NO/NO (without cAMP or Ca2+ response) ligands clustered. Except for fMLP-NGF and PAF-IFNb pairs of ligands all ligands this group look uncorrelated, without the explicit projection to the particular downstream clusters. (5.97 MB TIF) [file pone.0004189.s003.tif]

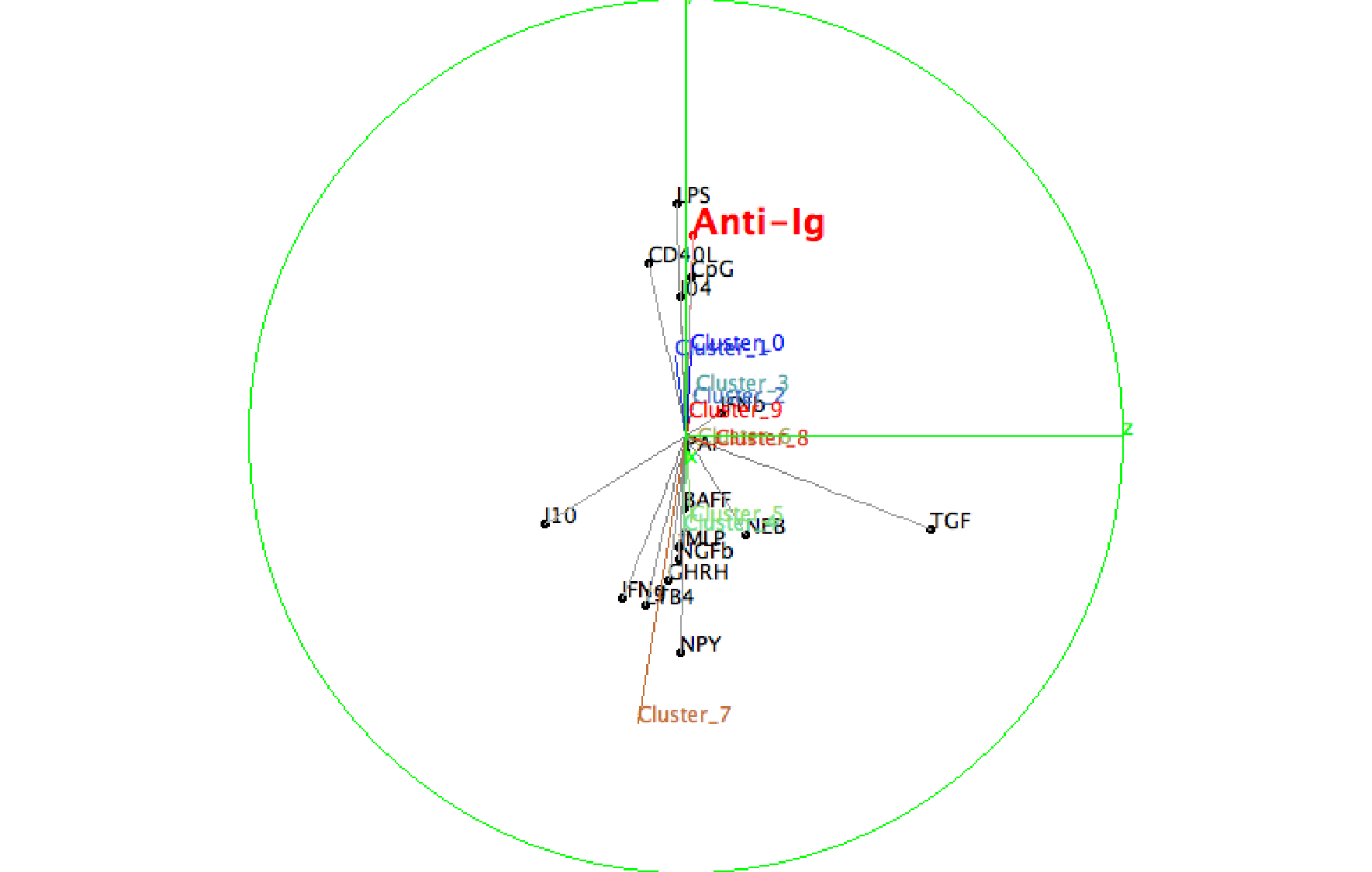

Supplement: Figure S2 — NO/NO ligands clustered together with AIG NO/YES (strongest Ca2+-inducing ligand). AIG is the closest to CD40, LPS, CpG and IL4 ligands with strong proliferate and differentiation response [5]. It is interesting to notice that adding AIG ligand made the picture of NO/NO ligands completely reorganized, thus brining those 5 ligands closely, while all others moved away. (5.97 MB TIF) [file pone.0004189.s004.tif]

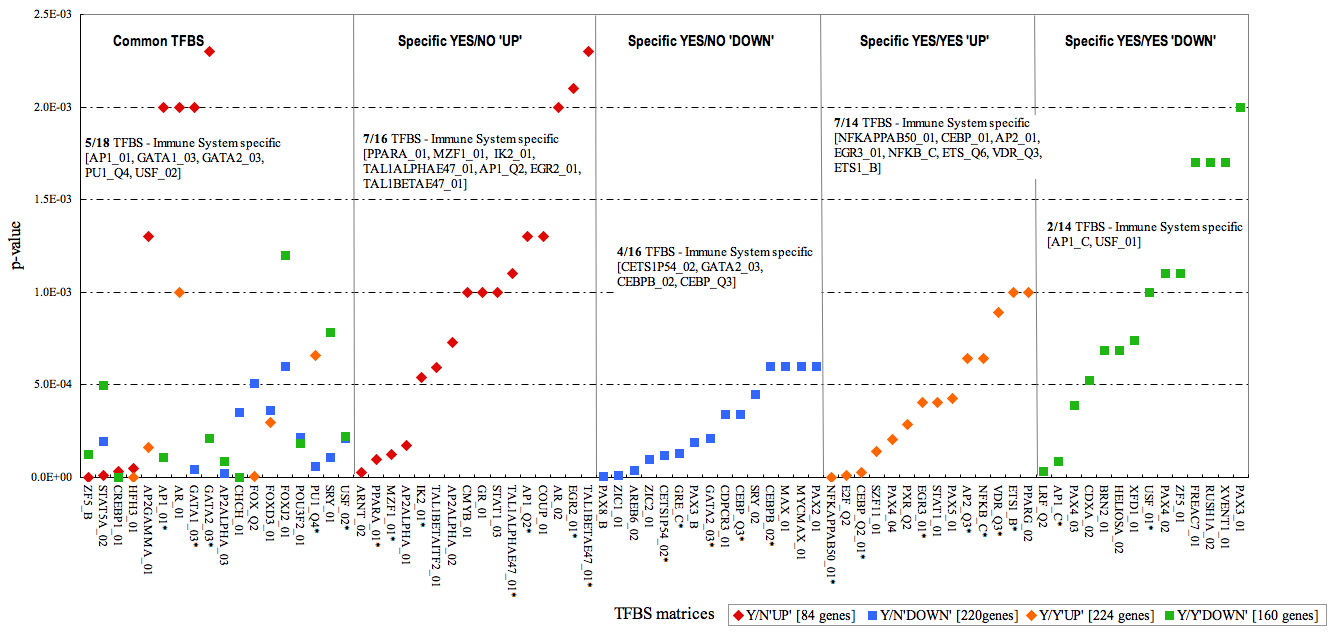

Supplement: Figure S3 — The common and specific transcription factor binding sites (TFBS) found in 4 sub-groups of YES/NO and YES/YES ligands groups of B-Cell. Horizontal axis lists the matrices names, which former parts (like ‘MZF1’ in MZF1_01) indicate the TFBS name and the latter parts (like ‘_01’ in MZF1_01) point to the experimental-base quality of the matrices in the descending order from ‘1’ to ‘6’. From the top 30 TFBS we selected matrices within the quality index range from 1 to 4. Asterisks* indicate known regulators of the Immune System, which constitutes 23% of the total number of TFBS (25/78) for B-Cell. The respective number of genes in the right-down corner indicates the numbers of genes which promoters were subjected to the transcriptional analysis and depicted in Figure 3 of the manuscript. (2.53 MB TIF) [file pone.0004189.s005.tif]

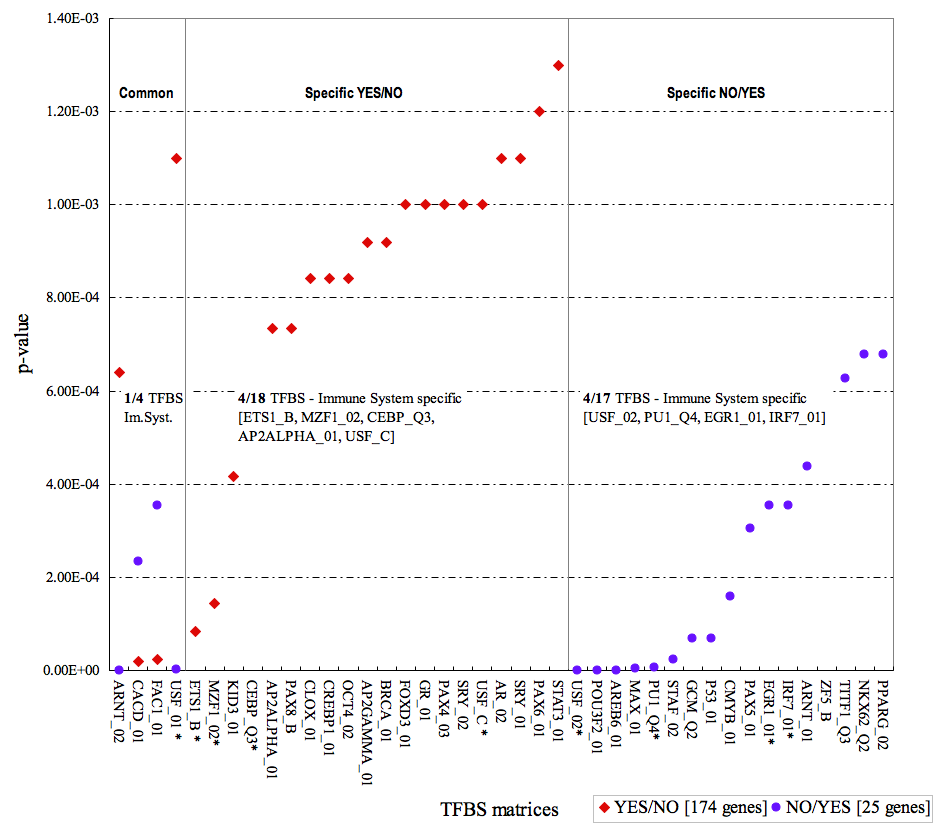

Supplement: Figure S4 — The common and specific transcription factor binding sites (TFBS) found in 2 YES/NO and NO/YES sub-regions of macrophage. Horizontal axis lists the matrices names, which former parts (like ‘MZF1’ in MZF1_01) indicate the TFBS name and the latter parts (like ‘_01’ in MZF1_01) point to the experimental-base quality of the matrices in the descending order from ‘1’ to ‘6’. From the top 30 TFBS we selected matrices within the quality index range from 1 to 4. Asterisks* indicate known regulators of the Immune System, which constitutes 23% of the total number of TFBS (9/39) selected for macrophage. The respective numbers of genes in the right-down corner indicate the numbers of genes which promoters were subjected to the transcriptional analysis and depicted in Figure 5 of the manuscript. (2.37 MB TIF) [file pone.0004189.s006.tif]
